# Prospective Evaluation of Radiotherapy-Induced Immunologic and Genetic Effects in Colorectal Cancer Oligo-Metastatic Patients with Lung-Limited Disease: The PRELUDE-1 Study

**DOI:** 10.3390/cancers13164236

**Published:** 2021-08-23

**Authors:** Alessandro Ottaiano, Angela Petito, Mariachiara Santorsola, Valerio Gigantino, Maurizio Capuozzo, Daniela Fontanella, Rossella Di Franco, Valentina Borzillo, Sergio Buonopane, Vincenzo Ravo, Esmeralda Scipilliti, Giuseppe Totaro, Marcello Serra, Gianluca Ametrano, Roberta Penta, Fabiana Tatangelo, Giosuè Scognamiglio, Annabella Di Mauro, Maurizio Di Bonito, Maria Napolitano, Stefania Scala, Giuseppina Rea, Sara Santagata, Angela Lombardi, Anna Grimaldi, Carlo Caputo, Anna Crispo, Egidio Celentano, Gianfranco De Feo, Luisa Circelli, Giovanni Savarese, Raffaella Ruggiero, Francesco Perri, Vincenza Granata, Gerardo Botti, Michele Caraglia, Guglielmo Nasti, Paolo Muto

**Affiliations:** 1 SSD—Innovative Therapies for Abdominal Metastases Unit, Istituto Nazionale Tumori di Napoli, IRCCS “G. Pascale”, via M. Semmola, 80131 Naples, Italy; mariachiara.santorsola@istitutotumori.na.it (M.S.); g.nasti@istitutotumori.na.it (G.N.); 2 Radiotherapy Unit, Istituto Nazionale Tumori di Napoli, IRCCS “G. Pascale”, via M. Semmola, 80131 Naples, Italy; angela.petito@istitutotumori.na.it (A.P.); r.difranco@istitutotumori.na.it (R.D.F.); valentina.borzillo@istitutotumori.na.it (V.B.); sergio.buonopane@istitutotumori.na.it (S.B.); v.ravo@istitutotumori.na.it (V.R.); esmeralda.scipilliti@istitutotumori.na.it (E.S.); giuseppe.totaro@istitutotumori.na.it (G.T.); marcello.serra@istitutotumori.na.it (M.S.); gianluca.ametrano@istitutotumori.na.it (G.A.); p.muto@istitutotumori.na.it (P.M.); 3 Innovalab Scarl, Molecular Biology, Centro Direzionale, Isola A2, 80143 Naples, Italy; valeriogigantino@gmail.com (V.G.); maurizio.capuozzo@gmail.com (M.C.); d.fontanella@tiscali.it (D.F.); 4 Oncohaematology Department, A.O.R.N. Santobono-Pausilipon di Napoli, 80123 Naples, Italy; robertapenta@inwind.it; 5 Pathology Unit, Istituto Nazionale Tumori di Napoli, IRCCS “G. Pascale”, via M. Semmola, 80131 Naples, Italy; f.tatangelo@istitutotumori.na.it (F.T.); giosue.scognamiglio@istitutotumori.na.it (G.S.); annabella.dimauro@istitutotumori.na.it (A.D.M.); m.dibonito@istitutotumori.na.i (M.D.B.); 6 Functional Genomics, Istituto Nazionale Tumori di Napoli, IRCCS “G. Pascale”, via M. Semmola, 80131 Naples, Italy; maria.napolitano@istitutotumori.na.it (M.N.); s.scala@istitutotumori.na.it (S.S.); g.rea@istitutoumori.na.it (G.R.); sara.santagata@istitutotumori.na.it (S.S.); 7 Department of Precision Medicine, University of Campania “L. Vanvitelli”, via de Crecchio 7, 80138 Naples, Italy; angelalombardi@hotmail.it (A.L.); grim.anna@tiscali.it (A.G.); carlo.caputo@unicampania.it (C.C.); Michele.caraglia@unicampania.it (M.C.); 8 Epidemiology and Biostatistics Unit, Istituto Nazionale Tumori di Napoli, IRCCS “G. Pascale”, via M. Semmola, 80131 Naples, Italy; a.crispo@istitutotumori.na.it (A.C.); e.celentano@istitutotumori.na.it (E.C.); 9 Scientific Directorate, Istituto Nazionale Tumori di Napoli, IRCCS “G. Pascale”, via M. Semmola, 80131 Naples, Italy; g.defeo@istitutotumori.na.it (G.D.F.); g.botti@istitutotumori.na.it (G.B.); 10 AMES, Centro Polidiagnostico Strumentale srl, 80013 Naples, Italy; lulacir@libero.it (L.C.); genetica@centroames.it (G.S.); raffaella.ruggiero@centroames.it (R.R.); 11 Head&Neck Unit, Istituto Nazionale Tumori di Napoli, IRCCS “G. Pascale”, via M. Semmola, 80131 Naples, Italy; f.perri@istitutotumori.na.it; 12 Radiology Unit, Istituto Nazionale Tumori di Napoli, IRCCS “G. Pascale”, via M. Semmola, 80131 Naples, Italy; v.granata@istitutotumori.na.it

**Keywords:** stereotactic radiation therapy, KRAS, abscopal effect, lung metastases, colorectal cancer

## Abstract

**Simple Summary:**

The management of advanced colorectal cancer (CRC) has been greatly improved with integrated strategies including stereotactic radiation therapy (SRT). It is a safe and effective option, particularly in oligo-metastatic (om) CRC patients. Interestingly, it has been demonstrated that SRT can induce regression of tumors in non-irradiated regions (“abscopal effect”) through stimulation of anti-tumor immune effects (“radiation-induced immunity”). We have recently shown that lung-limited omCRC is characterized by regression of tumor clones bearing specific key driver gene mutations. The aim of the PRELUDE-1 study is to assess the genetic and immunologic evolutions on tumor cancer/host cells induced by SRT in lung-limited omCRC through liquid biopsies and Next Generation Sequencing of tumor exome, HLA repertoire assessment, peripheral immune cells, and cytokine dynamics characterizations. An important secondary objective is the first prospective evaluation of the abscopal effect. The PRELUDE-1 results will help to identify subsets of patients more prone to show the abscopal effect. The PRELUDE-1 trial was registered into the clinicaltrials.gov registry on 22 April 2021, with identifier NCT04854213.

**Abstract:**

Background: in recent years, the management of advanced colorectal cancer (CRC) has been greatly improved with integrated strategies including stereotactic radiation therapy (SRT). The administration of SRT has been demonstrated, particularly in oligo-metastatic (om) CRC, to be a safe and effective option. Interestingly, it has been demonstrated that SRT can induce regression of tumors in non-irradiated regions (“abscopal effect”) through stimulation of anti-tumor immune effects (“radiation-induced immunity”). We have recently shown that lung-limited omCRC is characterized by regression of tumor clones bearing specific key driver gene mutations. Aims: to assess the genetic evolution on tumor cancer cells induced by SRT in lung-limited omCRC. Secondary objectives included descriptions of the abscopal effect, responses’ duration, toxicity, and progression-free survival. A translational research will be performed to evaluate tumor genetic evolution (through liquid biopsies and Next Generation Sequencing), HLA class I repertoire, peripheral immune cells, and cytokine dynamics. Methods: PRELUDE-1 is a prospective translational study. SRT will be administered only to the largest nodule (with a maximum diameter ≤ 25 mm) in omCRC with two or three radiologically evident lesions. The sample size is based on the innovative hypothesis that radiation-induced immunity could induce regression of tumor clones bearing KRAS oncogene mutations. According to the binomial test, considering the frequency of KRAS mutations and assuming a probability of mutant KRAS→wild type KRAS of p_0_ = 0.0077, with α = 0.05 and 1-β = 0.60, the final sample size is 25 patients.

## 1. Introduction

Colorectal cancer (CRC) represents the third most frequent cancer in both sexes. Unfortunately, more than 30% of patients are diagnosed with metastatic and unresectable disease mostly involving the liver [1]. Lungs and lymphnodes are also frequently targeted by metastatic CRC (mCRC). Chemotherapy (fluoropyirimidines, oxaliplatin, irinotecan) and biologic drugs (bevacizumab, aflibercept, cetuximab, panitumumab) have improved survival, which in rare cases can surpass 30 months [2]. Furthermore, in recent years, integrated strategies including stereotactic radiation therapy (SRT) have shown to be effective, particularly in oligo-metastatic CRC (omCRC) [3]. The omCRC clinical setting is represented by patients bearing indolent and low-burden mCRC. However, its definition is elusive and difficult. In fact, besides the tumor burden (≤3 lesions per organ with a total tumor size ≤7 cm) [4,5], it should also be considered the “rate of metastases development” [6,7,8]. However, to date, the immunologic and genetic characteristics underlying the oligo-metastatic phenotype are largely unknown and unexplored. We have recently shown that lung-limited omCRC is characterized by regressive mutations in key driver genes, suggesting that the oligo-metastatic status relies on atypical genetic evolution of cancer [9].

In recent years, the following considerations prompted the use of SRT in the treatment of omCRC: (i) the opportunity to delay the start of more toxic therapeutic approaches; (ii) the radio-sensitivity of colorectal cancer; (iii) the amelioration of the technique (sparing of healthy tissue and high and hypo-fractionated irradiation doses); and (iv) the increasing availability of the technique. 

## 2. Trial Rationale

### 2.1. Rationale for Evaluating the “Abscopal Effect” in omCRC

The “abscopal effect” (from Latin abscopus) is the anti-tumor effect elicited away from the target. In fact, high-dose radiotherapy (clinically used in hypo-fractionated regimens of SRT) can stimulate anti-tumor immune effects: “radiation-induced immunity” [10,11,12,13,14,15,16]. SRT is based on a great precision of tumor targeting allowing the use of high-doses in a hypo-fractionated manner. Oligo-metastatic cancers with controlled primary lesions are the ideal setting for SRT. Previous studies demonstrated that SRT is able to improve survival in lung-limited omCRC patients with median survivals approaching to three years. Significant prognostic factors are the number and the volume of lung metastases [17,18,19]. Unfortunately, the presence of occult microscopic tumor cell deposits at other sites is responsible for therecurrence and development of new distant metastases after SRT. Interestingly, hypo-fractionated SRT determines specific changes in the immune contexture of tumors characterized by the increase of human leukocyte antigen (HLA) class I expression, of antigen-presenting cells (APCs), of cytotoxic T lymphocytes (CTLs), of natural killer (NK) cells, and of interferon(IFN)-gamma secretion [20,21,22]. The last cytokine concurs to increase intracellular peptide levels through enhanced tumor antigens degradation. Hypo-fractionated radiation alsoinduces cell death by upregulating Fas and many other cytokines such as interleukin (IL)-2, IL-1α, IL-1β, IL-12, and tumor necrosis factor (TNF)-α involved in the initiation of an adaptive anti-tumor immune response [11,23,24,25,26,27,28]. These immunologic phenomena may be responsible for anti-tumor effects away from the irradiated target. Interestingly, a study by Lee et al. showed that the anti-tumor properties of high-dose radiotherapy associates with recruitment and priming of T cells in immune-competent mice; the same cancer was radio-resistant in immune-deficient or CD+ T-cell depleted animals [10]. 

### 2.2. Rationale for Evaluating Immune Regulatory Cells

Soluble mediators (cytokines, chemokines, etc.) and effector/regulatory lymphocytes participate in the orchestration of anti-tumor immunity through a dynamic balance between activation and inhibition of the immune response. In fact, many innovative drugs act by revitalizing the anti-tumor response [29]. The hypothesis that regulatory effectors can influence the extent of radiation-induced immunity deserves to be verified. The most important actors in the negative regulation of immune responses are: T regulatory cells (Tregs), myeloid-derived suppressor cells (MDSCs), and tumor-associated macrophages (TAMs) [30,31,32,33,34]. Interestingly, both MDSCs and Tregs increase in the peripheral blood (PB) of preclinical tumor models and suppress T-cell functions, which, in turn, favors cancer proliferation and metastases [35]. Tregs characterized as CD4+CD25+ T cells are fundamental in preserving self-tolerance and thus avoiding autoimmune activation [36,37,38,39,40,41,42]. They inhibit T cell activation through the secretion of immune-suppressive cytokines (IL-10, IL-35, TGF-beta, etc.) or surface expression of PD-L1 [41,42]. MDSCs are immature, myeloid cells able to inhibit the anti-tumor responses [43,44]. MDSCs secrete suppressive cytokines (e.g., IL-10, arginase, TGF-beta, etc.) that inhibit T cells and stimulate Tregs and M2 macrophages [33]. It was previously demonstrated that MDSCs and Tregs’ basal values are significantly increased in locally advanced rectal cancer (LARC) patients who received neoadjuvant chemo-radiotherapy before surgery versus healthy donors. Moreover, LARC-poor responder patients had a higher amount of Tregs in peripheral blood [45].

### 2.3. Human Leukocyte Antigens (HLA) and Anti-Cancer Immune Response

T cells’activation is based on sequential phases starting into specialized lymphoid organs. T cell precursors recognize tumor-derived peptide epitopes/HLA complexes on APCs through their T-cell receptors (TCRs) [46,47,48,49]. The binding between specific peptides processed by the proteasome system and specific HLA molecules occurs in the cytoplasm of APCs. Several algorithms have been developed to predict the binding affinity between epitopic sequences and HLA molecules [50,51,52,53,54]. In fact, polymorphisms of HLA-I alleles account for a different probability of tumor peptide recognition and subsequent activation by tumor-infiltrating T cells. Thus, patients with different class I and II HLA haplotypes have differential immune reactivity to the same tumor epitopes (CTL priming, expansion, and activation). For this reason, in the PRELUDE-1 study HLA haplotypes will be evaluated to adequately interpret eventual abscopal effects in lung-limited omCRC and to generate hypotheses on tumor-derived peptides’ repertoire.

### 2.4. Rationale for Evaluating Genetic Evolution of Cancer through Liquid Biopsies

The cancer genome is highly heterogeneous and dynamic. Mutation gains in some genes prompt cancer progression and resistance to therapy. Study of these changes is crucial to understand cancer biology and to propose innovative treatments. The most direct method to assess cancer genetics relies on the sampling of tumor tissues and its molecular characterization through whole genome sequencing techniques [55,56]. However, some issues can limit these evaluations since (i) biopsies are invasive and cannot be easily repeated during the treatments, (ii) tumor tissue is not always available, (iii) the genetic fingerprint of the neoplasia can change during the time due to the clonal selection of the host environment and to the different treatments. Alternatively, liquid biopsy is a non-invasive method to characterize cancer DNA, which can be repeated over time and which can collect circulating cancer material coming from all the clones of the neoplasia [57]. The technique is based on assessing tumor DNA released into the blood of cancer patients. In fact, it is well known that at least three cancer-associated phenomena concur to enrich the blood flow of tumor DNA: apoptosis, cell necrosis, and active secretion. Therefore, circulating tumor DNA (ctDNA) represents the tumor fingerprint in the blood. Next-generation sequencing (NGS), whose complete dissertation is beyond the scope of this study, is a suitable and reliable tool for genetic characterization through liquid biopsy in CRC [58,59]. 

Identifying the genetic and immunologic evolutions of cancer, in time and space, is a new and challenging issue. The introduction of high-throughput genetic assessments including NGS has enormously prompted this aspect of cancer research. Such an approach is providing novel and unexpected insights in cancer biology and suggesting new therapeutic strategies. Many studies in pmCRC have revealed heterogeneous results in the genetics of primary tumors (PT) and “matched” metastatic lesions (as evidenced by the occurrence of *de novo* mutations) with concordance rates (shared point mutations/total number of point mutations) ranging from 0 to 100% [60,61,62,63,64]. 

### 2.5. Definition of “Genetic Regression”

In a previous study in lung-limited omCRC, we documented changes of KRAS status in metastatic formalin-fixed and paraffin-embedded (FFPE) tumor tissues compared to the primary ones (KRAS was mutated in primary tumor but not in subsequent metastases) [9]. These genetic trajectories were associated with a good prognosis as observed also in omCRC with liver-limited disease [65] and poly-metastatic CRC [66]. Therefore, we defined “genetic regression” as the loss of the genetic alterations in a specific key-driver gene from the primary tumor to the metastatic one (i.e., mutant KRAS (mutKRAS) in primary tumor→wild-type KRAS (wtKRAS) in metastases). This issue was opposed to “progressive” genetic trajectory (i.e., wtKRAS in primary tumor→mutKRAS in metastases) where the metastatic lesions gain genetic alterations advantageous for the tumor development [67]. RAS proteins (specifically, KRAS and NRAS) are small GTPases along the epidermal growth factor receptor (EGFR) pathway [68,69]. When activated by ligand/receptor binding, RAS protein releases GTP, binds to GTP, and, in turn, activates crucial kinases(i.e., RAF proteins, phosphoinositide 3-kinase (PI3K)-AKT, etc.) involved in several cancer-related phenomena (migration, survival, adhesion, growth, and differentiation) [70]. These data indicate that omCRC tumor clones may follow regressive genetic trajectories differing from those of cancer cells with poly-metastatic behavior.

### 2.6. Genetic Regression of KRAS in an Immunologic Perspective 

The hypothesis on the elimination by the immune system of KRAS mutant clones is under intensive investigation by our and other groups. A complete description of factors involved in the interaction between immune and cancer cells is beyond the scope of this article. However, it has been demonstrated that T cells can recognize peptide epitopes presented by HLA class I on the surface of tumor cells. In fact, DNA mutations can lead to the formation of altered proteins and related peptides absent from the normal proteome. These peptides are called “neoantigens.” Many studies demonstrated that mutation-derived neoantigens can trigger specific tumor clone elimination [71,72,73]. Interestingly, in multiple myeloma, KRAS gene mutations were frequently found in highly immunogenic tumors [74], and, in CRC patients responding to adoptive T-cell therapy, neoantigens derived from mutated KRAS (p.G12D) and presented by HLA-C*08:02 were responsible for CRC cells’ recognition and elimination [75]. “Genetic regression” of KRAS can be hypothesized as an effect of KRAS-mutant neoantigens’ recognition.

## 3. Trial Design

### 3.1. Objectives

#### 3.1.1. Primary Objective

The primary objective is to verify that SRT can induce regression of KRAS mutant clones into the entire metastatic mass in a clean model of oligo-metastatic CRC. 

#### 3.1.2. Secondary Objectives

The most important secondary objective is the description of eventual radiologic responses in non-irradiated disease (abscopal effect). It will be measured through total-body computed tomography (CT) with iv contrast (if contraindicated: chest-computed tomography without iv contrast).

Other secondary objectives are the responses’ duration, progression-free survival (PFS), and toxicity (see beyond for response and toxicity assessment criteria). The response duration will be measured from the time of documented objective response until documented tumor progression. PFS will be determined from the data of treatment start until progression. Overall survival is generally long in this clinical setting so that it cannot be evaluated in the short time of the study. 

#### 3.1.3. Tertiary and Correlative Objectives 

Tertiary and correlative objectives consist in exploratory and descriptive studies of the tumor immune microenvironment in primary tumors, ^18^F-deoxyglucose positron emission tomography (FDG-PET) metabolic responses, HLA repertoire, and immune regulatory cells.

### 3.2. Design and Sample Size Calculation

PRELUDE-1 is a prospective, translational, and proof-of-principle study to detect regression of KRAS mutations in CRC induced by SRT. It will be conducted at the academic hospital Istituto Nazionale Tumori di Napoli, IRCCS “G. Pascale” in Naples (Italy). The sample size of the PRELUDE-1 study is based on the innovative hypothesis that radiation-induced immunity could induce regressive trajectories in metastases bearing KRAS oncogene mutations (i.e., mutKRAS→wtKRAS). 

According to the binomial test, considering a frequency of KRAS mutations of 50.0% (the study is not restricted to KRAS mutated patients) and assuming a probability of mutKRAS→wtKRAS of p_0_ = 0.0077 [9], with α = 0.05, 1-β = 0.60, p = the *a priori* success rate, and X = the number of patients with mutKRAS→wtKRAS, the final sample size is 25 patients. With the algorithm $P\left(X,N,p\right)=\left(\begin{array}{c} N\\ x \end{array}\right)p^{x}{\left(1-p\right)}^{n-x}$, the regressive trajectory is statistically relevant if mutations occurs in >2 out of the planned 25 patients.

### 3.3. Ethical Considerations

The protocol has been developed according to the Good Clinical Practice guidelines of the International Conference on Harmonization (ICH) and of the Declaration of Helsinki principles. The Ethical Committee of the National Cancer Institute of Naples, Italy approved it with document no. 88/20. Written informed consent will be obtained before starting the collection of blood samples and SRT. The structure that has the responsibility for registration, collection, and management of personal data will protect the privacy of patients included in the PRELUDE-1 study. A progressive numerical code will be attributed to the patients to manage documents, electronic data systems, or communications. A list with numerical codes and associated patients’ names will be kept exclusively at the secretariat of the PRELUDE-1 study. Only the Ministry of Health or Ethics Committees can know patients’ names (as required by the current legislation) for inspection and control purposes. 

The choice to treat two or three nodules with SRT was based on scientific, practical, and ethical considerations that deserve to be briefly described. The mainstay treatment of one nodule is surgery; moreover, abscopal effect cannot be detected in this context. On the other hand, four nodules are not consistent with the definition of oligo-metastatic disease we adopted [4,5]; in this case, a front-line systemic treatment is more appropriate. However, SRT will be applied only if the largest nodule measures ≤25 mm and the sum of all nodules is ≤7 cm. A strict monitoring of non-irradiated nodules will be performed already after two months in order to treat those with SRT in case of progression. However, chemotherapy is allowed at any time after the first radiologic assessment and liquid biopsy post-SRT according to multidisciplinary discussion and the European Society of Medical Oncology (ESMO) guidelines. The independent ethical committee approved the protocol after reassurance on these specific aspects.

### 3.4. Liquid Biopsy and Sequencing

For liquid biopsies, blood samples will be collected in streck-cell-free DNA BCT^®^ (pluriSelect Life Science, DeutscherPlatz 5c, 04103 Leipzig, DE) tubes and centrifuged at 1800× *g* for 10min. Thereafter, plasma will be further centrifuged at 3000× *g* for 10min, aliquoted, and stored at −80°C prior to analysis. The QIAamp DNA Blood Mini Kit (Qiagen, Hilden, Germany) will be used to extract circulating free (cfDNA). Formalin-fixed and paraffin-embedded (FFPE) tissue specimens of primary lesions will be collected and ten μM serial sections cut from each tissue specimen for microdissection of tumor cells under morphological control. DNA isolation will be performed through the MGF03-Genomic DNA FFPE One-Step Kit (MagCoreDiatech, RBCBioscience, Ln. 235, Baoqiao Rd., Xindian Dist., New Taipei City 23145, Taiwan). Tumor DNA quality from PEFF tissues will be established using the FFPE QC Kit according to the manufacturer’s protocol (Illumina, San Diego, CA, USA). Concentration, quantity, and integrity of cfDNA will be assessed through a 2100 BioAnalyzer (Agilent Technologies, Santa Clara, CA, USA) to determine the size distribution of cfDNA fragments. DNA libraries for tumor genetic evaluations will be obtained with TruSigt Oncology (TSO) 500 kit, based on gene-target enrichment that analyzes 523 cancer-relevant genes (see Supplementary File for the complete list of genes). The assay reveals small nucleotide variants (SNVs), indels, splice variants, as well as tumor mutational burden (TMB) and microsatellite instability (MSI) as immunotherapy biomarkers. Sequencing will be performed on an Illumina NovaSeq 6000 (San Diego, CA, USA) platform. 

### 3.5. Tumor-Infiltrating Lymphocytes Analysis in Primary CRC

Immunohistochemistry (IHC) will be applied to analyze T cell subsets in the tumor microenvironment. Briefly, formalin-fixed, paraffin-embedded 4-μm tissue sections of primary CRC will be stained according to a biotin-streptavidin-peroxidase method (YLEM kit, Rome, Italy) previously described [9]. Primary antibodies to characterize lymphocyte subpopulations will be anti-human CD3, anti-human CD8, anti-human FoxP3, and anti-human Granzyme B [76]. Finally, immune-stained slides will be counterstained with hematoxylin, dehydrated, and mounted in Diatex. Substituting the primary antibody with a mouse myeloma protein of the same subclass at the same concentration as the monoclonal antibody will represent the negative controls. An automated scanning microscope and image analysis system (Genetix, San Jose, CA) will be used to scan the slides. Two expert pathologists (G.B. and F.T.) blinded to all clinical information will review all qualitative and quantitative analyses of T-cell subsets. The tumor contexture will be divided into tumor core (TC) and invasive margins (IM) as previouslydescribed [77]. T-cell subsets densities (cells/mm^2^) results will be depicted through the arithmetic averages + 2 standard deviations (SD). 

### 3.6. HLA Allele Haplotype Determination

HLA haplotypes at loci A, B, C, and DRB1 will be assessed centrally at the University of Campania “L. Vanvitelli” through a low-medium-resolution reverse SSO DNA typing assays (One-Lambda Luminex Technology LABScan 100, HLA Fusion Software) on genomic DNA extracted according to the kit manufacturer from whole blood or peripheral blood mononuclear cells. Comparisonof the results with our database bank including healthy bone marrow donors (Campania National Registry) will be performed.

### 3.7. Cytokines Determination

Blood samples will be collected from a peripheral vein at the decided times during the treatment courses and kept on ice. Serum will be aliquoted and stored at −80 °C until analyzed after centrifugation (3000 r.p.m. for 10 min at 4 °C). An ELISA-based immunoassay, using monoclonal antibodies specific for the different target proteins will be used. Multiple soluble cytokines will be assessedwith either commercially or customized kits: Interferon (IFN)γ, TNFα, IL-4, IL-8, IL-10, IL-12, IL-17, vascular endothelial growth factor (VEGF), granulocyte colony-stimulating factor (G-CSF), angiopoietin-2, and hegapocyte growth factor(HGF). Each experiment will be performed in duplicate. Serum levels of all proteins will be determined using a multiplate reader (Biorad, Hercules, CA, USA), and the substance concentration will be assessedthrougha standard curve, with software provided by the manufacturer (BioRad Manager Software, Hercules, CA, USA).

### 3.8. Bioinformatics Analysis and Data Presentation

An IlluminaTruSigth Oncology bioinformatics pipeline will be used to analyze DNA sequencing results. Number of reads and coverage in the target regions will be required to be above the manufacturer’s suggested thresholds. Sequence data will be aligned to the human reference genome GRCh37 (http://www.ncbi.nlm.nih.gov/projects/genome/assembly/grc/human/index.shtml) (accessed on 22 June 2021) through the Burrows–Wheeler Aligner with default parameters [78]. GENCODE, ICGC-PCAWG, dbNSFP, COSMIC, ClinVar, CancerMine, 1000Genomes, OncoScore, CIViC, and CBMDB databases will be used to assess the clinical significance of the found variants. The global minor allele frequency cut-off to filter and remove variants will be <1%. The four-tiered structure, designed by the joint consensus recommendation of the AMP/ACMG [79] will be applied to prioritize variants. However, to exclude residual false positives, variants will be also manually curated. The overall genetic evolution will be indicated with: (i) the percent of mutational concordance between different samples and (ii) Venn diagrams in order to plot intersections among genetic results in different samples (see beyond). Phenolyzer, a computational tool that prioritizes genes on the basis of updated existing knowledge (protein–protein interactions, the sharing of biological pathways or gene families, gene–gene transcriptional regulation, etc.), will be used to evidence relevance and relationships between any “seed” genetic variants and “secondary” ones [80]. 

### 3.9. Eligibility Criteria

Inclusion criteria.

1. Cytological or histological diagnosis of colorectal adenocarcinoma.
2. Two or three asymptomatic lung metastases smaller than or equal to 25 mm.
3. Age <80 years.
4. ECOG performance status of 0 or 1.
5. Available FFPE resected primary tumor.
6. Negative pregnancy test for all potentially childbearing women.
7. Written informed consent.

Exclusion criteria.

1. Previous systemic anti-tumor treatments or radiotherapy interrupted at least 6 months before.
2. Neutrophils <2000/mm^3^ or platelets <100.000/mm^3^ or hemoglobin <9 g/dL; serum creatinine level> 1.5 times the maximum normal value; bilirubin level >3 times the maximum normal value; AST and/or ALT >5 times the maximum normal value.
3. Previous or concomitant malignant neoplasms (excluding basal or spinocellular cutaneous carcinoma or in situ carcinoma of the uterine cervix).
4. Active or uncontrolled infections.
5. Cardiovascular diseases including coronary artery disease (CAD), inadequately controlled hypertension, ischemic or hemorrhagic stroke, moderate/sever arrhythmias, aortic aneurysm requiring surgical repair, recent deep vein thrombosis with or without pulmonary embolisms, moderate/sever valvular heart diseases, recent arterial thrombosis.
6. Other uncontrolled or uncompensated diseases (diabetes, asthma, chronic obstructive pulmonary disease, etc.).
7. Refusal or inability to provide informed consent.
8. Impossibility to guarantee follow-up.

### 3.10. Radiotherapy Schedule

SRT will be administered according to a risk-adapted protocol; size and location of the tumor will drive doses and fractionations (54 Gy/3 fractions, 55 Gy/5 fractions, or 60 Gy/8 fractions). Regardless of the dose-fractionation regimen, the treatment will be delivered on alternate days. For all patients, a 4-D CT simulation scan will be acquired. Respiratory gating will be used in cases with thorax motion > 7 mm in any direction. The gross tumor volume (GTV) will be defined as the tumor visible on CT and PET imaging, and an internal GTV will encompass the GTV from all phases of respiration. A planning target volume (PTV) margin of 5 mm will be applied. The prescription point will be approximately the 80% isodose line surrounding the PTV, with the requirement that 95% of the PTV will be covered by 100% of the prescription dose. 

### 3.11. Timing of Exams and Procedures

**Figure 1:**
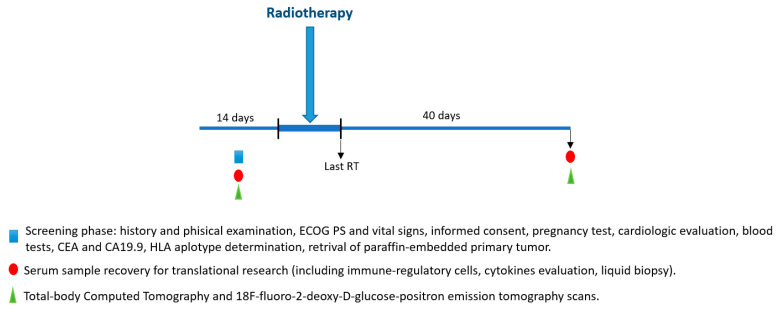
Timeline of PRELUDE-1 study.

Timings and types of procedures are synthesized in Figure 1 and Table 1.

Screening phase. Inclusion and exclusion criteria will be evaluated during the screening phase.

Baseline phase. Thereafter, after signing the informed consent, a venous blood sample (10 mL) for the liquid biopsy (LB) will be collected coinciding with the routine assessment before the SRT start. Baseline visit (clinical history, clinical examination, PS ECOG, vital signs) and exams (including basal LB (LB-T1)) will also be carried out within 14 days before the SRT start. Blood count and clinical biochemistry will be performed at our local laboratories. The following parameters will be assessed: blood count with leukocyte formula, hemoglobin, platelets, total bilirurbin, AST, ALT, alkaline phosphatase, serum creatinine, total proteins, sodium, potassium, calcium, urea, lactic dehydrogenase, creatinine clearance, CEA, and CA19.9. Patient’s heart rhythm and conduction will be evaluated with ECG. The ventricular ejection fraction will be assessed with cardiac ultrasonography.All potentially childbearing women musthave a negative pregnancy test during the screening phase. If requested by the local ethics committee, the test can also be repeated during treatment. Barrier methods for anti-conception must be used by all patients throughout the duration of the study. FDG-PET and total-body computed tomography with iv contrast or, if contraindicated, magnetic resonance (MRI) abdomen and high-definition chest-computed tomography without iv contrast will be performed. 

Treatment phase. Clinical examination and evaluation of vital signs will be performed at each radiotherapy visit. Cardiological evaluation, CEA, CA19.9, and assessment of response to treatment will be performed after 40 days and 2 months from the radiotherapy start and, thereafter, every 3 months until progression. 

“Interval” phase. LB-T2 will be performed 40 days after major nodule irradiation coinciding with post-RT cell blood count and biochemical assessment along with CT total-body-scan and FDG-PET. Thereafter, the disease will be monitored every three months, and non-irradiated nodules will be treated in case of volumetric progression according to clinical practice in oligo-metastatic disease.

End-of-study phase. The study ends after LB-T2 assessment. 

Follow-up. Additional radiotherapy, surgery, or systemic treatment lines in case of appearance of new nodules or specific follow-up procedures will be applied at the discretion of the clinician responsible for the medical treatment. 

### 3.12. Response and Toxicity Assessment

Response will be assessed through total-body computed tomography (CT) with iv contrast (if contraindicated: abdomen MRI and high-definition chest-computed tomography without iv contrast), CEA, CA19.9, and PET-FDG after 40 days from the end of SRT. A CT scan will be repeated at two months and, thereafter, every three months until progression. RECIST v. 1.1 (https://recist.eortc.org/recist-1-1-2/) (accessed on 22 June 2021) will be applied to classify responses. An independent and blinded data monitoring committee (DMC) will review radiologic responses. The DMC will also review the PFS data. Toxicity will be evaluatedaccording to the CTCAE, version 4.0 (https://www.eortc.be/services/doc/ctc/CTCAE_4.03_2010-06-14_QuickReference_5x7.pdf) (accessed on 22 June 2021). The presence of eventual adverse events will be evaluated at each study visit. Patients will also notify the investigator by telephone in case of adverse events occurring between one visit and the next. The maximum grade per patient will be reported for each adverse event. A toxic effect of any grade on multiple occasions will be counted only once.

FDG-PET will be performed after 40 days from the treatment end to early monitor untreated nodules. In this case, tumor metabolic changes will be evaluated according to the European Organization for Research and Treatment of Cancer (EORTC) criteria [81,82]. Metabolic responses by FDG-PET/CT are defined as follows:

- Complete metabolic response (CMR), complete resolution of all metabolically active target and non-target lesions, and no new lesions;
- Partial metabolic response (PMR), 20% or greater decrease in SUV of target lesions with or without decrease in number/size of nontarget lesions, and no new lesions;
- Progressive metabolic disease (PMD), one or more new lesions, 20% or greater increase in SUV of target lesions, and/or unequivocal increase in FDG activity of nontarget lesions;
- Stable metabolic disease (SMD): not qualifying as CMR, PMR, or PMD.

### 3.13. Data Management

Clinical data will be registered in electronic case report forms (eCRFs). From a formal point of view, institutional standard operating procedures (SOPs) have been used to write and present this protocol to the ethical committee. Patient safety and integrity of the data are guaranteed by the strict adherence to SOPs (integral SOPs can be requested to monitoraggioscc@istitutotumori.na.it). Audits and other monitoring procedures will be planned by the Director of Scientific Monitoring and Research Quality Assurance (Dr. Gianfranco De Feo). The Principal Investigator will be responsible to clarify or respond to any eventual queries coming from the sanitary authorities or any component of the scientific community. The clinical database will be locked and analyzed at the end of enrollment. 

### 3.14. Patients’ Study Withdrawals

Patients’ withdrawals from study will be registered in eCRFs. The information will include the date and the reason for cessation. However, considering the high translational nature, the short duration of the study, its small sample size, and the good safety profile of SRT, withdrawals are expected to be extremely low. Patients off study therapy will be followed-up until death. Patients stopping the treatment early for any reason will not be excluded from the progression-free survival analysis; all patients will be included in the descriptive statistics.

### 3.15. Data Dissemination

Final and “interim” results of the study will be shown at national and international congresses. Final results will be submitted for publication to open access peer-reviewed scientific journals. Furthermore, upon Scientific Director authorization, the results will be disseminated by press releases by the Istituto Nazionale Tumori di Napoli, IRCCS “G. Pascale.”

## 4. Discussion

First described in 1953 [83], in recent years, some evidence has suggested that the abscopal effect of SRT may depend on immune system modulation [84]. However, despite an intriguing background and its potential application in cancer therapy, there are no prospective studies specifically addressing the biological and genetic phenomena underlying the “abscopal effect” (if any) of high-dose and hypo-fractionated radiotherapy. This is the most important aim of the PRELUDE-1 study.

The hypothesis that SRT could induce genetic regressive trajectories in omCRC is fascinating and deserves a scientific effort to be verified. Therefore, we included in the translational section of the PRELUDE-1 study the assessment of genetic trajectories of KRAS, which is also the primary objective of the study and that is the main reason for its sample size. The dynamism of tumor clones in space and time is a crucial issue for future research in oncology. The main forces influencing the molecular and biologic shape of neoplastic populations are still largely unknown. Is the selection of tumor clones on stochastic/evolutionistic pushing? Does immunologic selection concur to modify the neoplastic population during progression or treatments? Oligo-metastatic disease may provide an interesting clinical model to study some aspects of these questions. We previously found that “regressive genetic trajectories” of KRAS, a key driver gene, could account for the good prognosis in the omCRC setting, and we are evaluating the hypothesis that an immunological negative selection of KRAS-mutated aggressive tumor clones could be responsible for this effect.

It has already been described that, in addition to direct damage to neoplastic cells, a radiation-induced immunity, based on HLA expression, APC and CTLs recruitment and activation, and cytokines release, exists. Some of these immunologic effects become systemic and potentially responsible for anti-tumor effects on the entire neoplastic progeny. In this context, the study of regulatory lymphocytes is of paramount interest. Notably, MDSCs and Tregs can suppress anti-tumor immunity, and their assessment could help to identify subsets of patients more prone to show the abscopal effect. Study of the HLA-I genotype although exploratory will help to interpret the probability of both neo-antigen and immune recognition of tumors in case of the abscopal effect. 

## 5. Conclusions

PRELUDE-1 is the first prospective and translational study that, with its mono-institutional nature, will contribute to describe and clarify biologic and immunologic correlates of the SRT-induced abscopal effect through an innovative and high-throughput analytical approach. 

## Figures and Tables

**Table 1:** Schedule of assessments for PRELUDE-1 study.

Study Assessments	Within 14 Days	SRT Start	40 Days after Last SRT	After Two Months	Every Three Months	Follow-up
Informed consent	X					
Eligibility crietria	X					
Pregnancy test	X			X	X	X
Concurrent medications	X			X	X	X
Cardiologic evaluation	X			X	X	X
Anamnesis	X			X	X	X
Height	X					
Weight	X		X	X	X	X
Clinical examination, PS ECOG, vital signs	X		X	X	X	X
Blood count and clinical biochemistry, CEA, and CA19.9	X		X	X	X	X
Assessment of cytokines, regulatory cells, HLA haplotype determination	X		X			
Total-body computed tomography with iv contrast	X		X	X	X	X
^18^F-fluoro-2-deoxy-D glucose-positron emission tomography scans	X		X			
Liquid biopsy	X		X			
Adverse events evaluation	X		X	X	X	X
Progression-free survival				X	X	X

## Data Availability

All protocol data are reported in the manuscript.

## Supplementary Materials

The following are available online at https://www.mdpi.com/article/10.3390/cancers13164236/s1, supplementary file: List of studied genes through TSO 500

## References

[1] Siegel R., Naishadham D., Jemal A. (2012). Cancer statistics, 2012. CA Cancer J. Clin..

[2] Nappi A., Berretta M., Romano C., Tafuto S., Cassata A., Casaretti R., Silvestro L., De Divitiis C., Alessandrini L., Fiorica F. (2018). Metastatic Colorectal Cancer: Role of Target Therapies and Future Perspectives. Curr. Cancer Drug Targets.

[3] Lancia A., Ingrosso G., Carosi A., Di Murro L., Giudice E., Cicchetti S., Morelli P., Di Cristino D., Bruni C., Murgia A. (2017). Oli-gometastatic cancer: Stereotactic ablative radiotherapy for patients affected by isolated body metastasis. Acta Oncol..

[4] Rusthoven K.E., Kavanagh B.D., Burri S.H., Chen C., Cardenes H., Chidel M.A., Pugh T.J., Kane M., Gaspar L.E., Schefter T.E. (2009). Multi-Institutional Phase I/II Trial of Stereotactic Body Radiation Therapy for Lung Metastases. J. Clin. Oncol..

[5] Hellman S., Weichselbaum R.R. (1995). Oligometastases. J. Clin. Oncol..

[6] Niibe Y., Chang J.Y., Onishi H., Salama J., Hiraki T., Yamashita H. (2014). Oligometastases/oligo-recurrence of lung cancer. Pulm. Med..

[7] Withers H.R., Lee S. (2006). Modeling Growth Kinetics and Statistical Distribution of Oligometastases. Semin. Radiat. Oncol..

[8] Lussier Y.A., Khodarev N.N., Regan K., Corbin K., Li H., Ganai S., Khan S.A., Gnerlich J.L., Darga T.E., Fan H. (2012). Oligo -and polymetastatic progression in lungmetastasis(es) patients is associated with specific MicroRNAs. PLoS ONE.

[9] Ottaiano A., Circelli L., Lombardi A., Scala S., Martucci N., Galon J., Buonanno M., Scognamiglio G., Botti G., Hermitte F. (2020). Genetic trajectory and immune microenvironment of lung-specific oligometastatic colorectal cancer. Cell Death Dis..

[10] Lee Y., Auh S.L., Wang Y., Burnette B., Meng Y., Beckett M., Sharma R., Chin R., Tu T., Weichselbaum R.R. (2009). Therapeutic effects of ablative radiation on local tumor require CD8+ T cells: Changing strategies for cancer treatment. Blood.

[11] Lugade A.A., Sorensen E.W., Gerber S.A., Moran J.P., Frelinger J.G., Lord E.M. (2008). Radiation-induced IFN-gamma production within the tumor microenvironment influences antitumor immunity. J. Immunol..

[12] Hennel R., Brix N., Seidl K., Ernst A., Scheithauer H., Belka C., Lauber K. (2014). Release of monocyte migration signals by breast cancer cell lines after ablative and fractionated γ-irradiation. Radiat. Oncol..

[13] Postow M.A., Callahan M.K., Barker C.A., Yamada Y., Yuan J., Kitano S., Mu Z., Rasalan T., Adamow M., Ritter E. (2012). Immunologic Correlates of the Abscopal Effect in a Patient with Melanoma. N. Engl. J. Med..

[14] Golden E.B., Demaria S., Schiff P., Chachoua A., Formenti S.C. (2013). An Abscopal Response to Radiation and Ipilimumab in a Patient with Metastatic Non–Small Cell Lung Cancer. Cancer Immunol. Res..

[15] Gulley J., Arlen P.M., Bastian A., Morin S., Marte J., Beetham P., Tsang K.-Y., Yokokawa J., Hodge J.W., Ménard C. (2005). Combining a Recombinant Cancer Vaccine with Standard Definitive Radiotherapy in Patients with Localized Prostate Cancer. Clin. Cancer Res..

[16] Filatenkov A., Baker J., Mueller A.M., Kenkel J., Ahn G.-O., Dutt S., Zhang N., Kohrt H., Jensen K., Dejbakhsh-Jones S. (2015). Ablative Tumor Radiation Can Change the Tumor Immune Cell Microenvironment to Induce Durable Complete Remissions. Clin. Cancer Res..

[17] Filippi A., Guerrera F., Badellino S., Ceccarelli M., Castiglione A., Guarneri A., Spadi R., Racca P., Ciccone G., Ricardi U. (2016). Exploratory Analysis on Overall Survival after Either Surgery or Stereotactic Radiotherapy for Lung Oligometastases from Colorectal Cancer. Clin. Oncol..

[18] Pasqualetti F., Montrone S., Vivaldi C., Zani M., Fedele D., Fornaro L., Pasqualetti G., Salvatore L., Manfredi B., Laliscia C. (2017). Stereotactic Body Radiotherapy in Patients with Lung Oligometastases from Colorectal Cancer. Anticancer. Res..

[19] Agolli L., Bracci S., Nicosia L., Valeriani M., De Sanctis V., Osti M.F. (2017). Lung Metastases Treated with Stereotactic Ablative Radiation Therapy in Oligometastatic Colorectal Cancer Patients: Outcomes and Prognostic Factors After Long-Term Follow-Up. Clin. Colorectal Cancer.

[20] Siva S., MacManus M., Martin R.F., Martin O.A. (2015). Abscopal effects of radiation therapy: A clinical review for the radiobiologist. Cancer Lett..

[21] Sauter B., Albert M.L., Francisco L., Larsson M., Somersan S., Bhardwaj N. (2000). Consequences of cell death: Exposure to necrotic tumor cells, but not primary tissue cells or apoptotic cells, induces the maturation of immunostimulatory dendritic cells. J. Exp. Med..

[22] Todryk S., Melcher A., Hardwick N., Linardakis E., Bateman A., Colombo M.P., Stoppacciaro A., Vile R.G. (1999). Heat shock protein 70 induced during tumor cell killing induces Th1 cytokines and targets immature dendritic cell precursors to enhance antigen uptake. J. Immunol..

[23] Burnette B.C., Liang H., Lee Y., Chlewicki L., Khodarev N.N., Weichselbaum R.R., Fu Y.-X., Auh S.L. (2011). The Efficacy of Radiotherapy Relies upon Induction of Type I Interferon–Dependent Innate and Adaptive Immunity. Cancer Res..

[24] Lim J.Y.H., Gerber S.A., Murphy S.P., Lord E.M. (2014). Type I interferons induced by radiation therapy mediate recruitment and effector function of CD8+ T cells. Cancer Immunol. Immunother..

[25] Sharabi A.B., Nirschl C.J., Kochel C.M., Nirschl T.R., Francisca B.J., Velarde E., Deweese T.L., Drake C.G. (2015). Stereotactic Radiation Therapy Augments Antigen-Specific PD-1–Mediated Antitumor Immune Responses via Cross-Presentation of Tumor Antigen. Cancer Immunol. Res..

[26] Lugade A.A., Moran J.P., Gerber S.A., Rose R.C., Frelinger J.G., Lord E.M. (2005). Local Radiation Therapy of B16 Melanoma Tumors Increases the Generation of Tumor Antigen-Specific Effector Cells That Traffic to the Tumor. J. Immunol..

[27] Shiraishi K., Ishiwata Y., Nakagawa K., Yokochi S., Taruki C., Akuta T., Ohtomo K., Matsushima K., Tamatani T., Kanegasaki S. (2008). Enhancement of Antitumor Radiation Efficacy and Consistent Induction of the Abscopal Effect in Mice by ECI301, an Active Variant of Macrophage Inflammatory Protein-1α. Clin. Cancer Res..

[28] Khan M.A., Van Dyk J., Yeung I.W.T., Hill R.P. (2003). Partial volume rat lung irradiation; assessment of early DNA damage in different lung regions and effect of radical scavengers. Radiother. Oncol..

[29] Dyck L., Mills K.H. (2017). Immune checkpoints and their inhibition in cancer and infectious diseases. Eur. J. Immunol..

[30] Piccirillo C.A., Shevach E.M. (2004). Naturally occurring CD4+CD25+ immunoregulatory T cells: Central players in the arena of pe-ripheral tolerance. Semin. Immunol..

[31] Gabrilovich D.I. (2017). Myeloid-Derived Suppressor Cells. Cancer Immunol. Res..

[32] Huang B., Pan P.Y., Li Q., Sato A.I., Levy D.E., Bromberg J., Divino C.M., Chen S.H. (2006). Gr-11CD1151 immature myeloid suppressor cells mediate the development of tumor induced T regulatory cells and T-cell energy in tumor-bearing host. Cancer Res..

[33] Marvel D., Gabrilovich D.I. (2015). Myeloid-derived suppressor cells in the tumor microenvironment: Expect the unexpected. J. Clin. Investig..

[34] Gabrilovich D.I., Ostrand-Rosenberg S., Bronte V. (2012). Coordinated regulation of myeloid cells by tumours. Nat. Rev. Immunol..

[35] Lindau D., Gielen P., Kroesen M., Wesseling P., Adema G.J. (2013). The immunosuppressive tumour network: Myeloid-derived suppressor cells, regulatory T cells and natural killer T cells. Immunol..

[36] Sakaguchi S., Sakaguchi N., Asano M., Itoh M., Toda M. (1995). Pillars article: Immunologic self-tolerance maintained by activated T cells expressing IL-2 receptor α-chains (CD25). Breakdown of a single mechanism of self-tolerance causes various autoimmune diseases. J. Immunol..

[37] Sakaguchi S., Miyara M., Costantino C., Hafler D.A. (2010). FOXP3+ regulatory T cells in the human immune system. Nat. Rev. Immunol..

[38] Sakaguchi S., Yamaguchi T., Nomura T., Ono M. (2008). Regulatory T Cells and Immune Tolerance. Cell.

[39] Miyara M., Sakaguchi S. (2007). Natural regulatory T cells: Mechanisms of suppression. Trends Mol. Med..

[40] Hori S., Nomura T., Sakaguchi S. (2003). Control of Regulatory T Cell Development by the Transcription Factor Foxp3. Science.

[41] Raimondi G., Shufesky W.J., Tokita D., Morelli A.E., Thomson A.W. (2006). Regulated Compartmentalization of Programmed Cell Death-1 Discriminates CD4+CD25+ Resting Regulatory T Cells from Activated T Cells. J. Immunol..

[42] Togashi Y., Shitara K., Nishikawa H. (2019). Regulatory T cells in cancer immunosuppression—Implications for anticancer therapy. Nature.

[43] Ostrand-Rosenberg S., Sinha P. (2009). Myeloid-Derived Suppressor Cells: Linking Inflammation and Cancer. J. Immunol..

[44] Bronte V., Brandau S., Chen S.-H., Colombo M.P., Frey A.B., Greten T.F., Mandruzzato S., Murray P.J., Ochoa A., Ostrand-Rosenberg S. (2016). Recommendations for myeloid-derived suppressor cell nomenclature and characterization standards. Nat. Commun..

[45] Napolitano M., D’Alterio C., Cardone E., Trotta A.M., Pecori B., Rega D., Pace U., Scala D., Scognamiglio G., Tatangelo F. (2015). Peripheral myeloid-derived suppressor and T regulatory PD-1 positive cells predict response to neoadjuvant short-course ra-diotherapy in rectal cancer patients. Oncotarget.

[46] Wölfel T., Klehmann E., Müller C., Schütt K.H., Meyer zum Büschenfelde K.H., Knuth A. (1989). Lysis of human melanoma cells by autologous cytolytic T cell clones. Identification of human histocompatibility leukocyte antigen A2 as a restriction element for three different antigens. J. Exp. Med..

[47] Hunt D.F., Henderson R.A., Shabanowitz J., Sakaguchi K., Michel H., Sevilir N., Cox A.L., Appella E., Engelhard V.H. (1992). Characteri-zation of peptides bound to the class I MHC molecule HLA-A2.1 by mass spectrometry. Science.

[48] Crowley N.J., Darrow T.L., Quinn-Allen M., Seigler H.F. (1991). MHC-restricted recognition of autologous melanoma by tumor-specific cytotoxic T cells. Evidence for restriction by a dominant HLA-A allele. J. Immunol..

[49] McDonnell A.M., Robinson B.W.S., Currie A.J. (2010). Tumor Antigen Cross-Presentation and the Dendritic Cell: Where it All Begins?. Clin. Dev. Immunol..

[50] Falk K., Rötzschke O., Stevanovié S., Jung G., Rammensee H.-G. (1991). Allele-specific motifs revealed by sequencing of self-peptides eluted from MHC molecules. Nat. Cell Biol..

[51] Parker K., Bednarek M., E Coligan J. (1994). Scheme for ranking potential HLA-A2 binding peptides based on independent binding of individual peptide side-chains. J. Immunol..

[52] Rammensee H.-G., Bachmann J., Emmerich N.P.N., Bachor O.A., Stevanović S. (1999). SYFPEITHI: Database for MHC ligands and peptide motifs. Immunogenetics.

[53] Parmiani G., Russo V., Maccalli C., Parolini D., Rizzo N., Maio M. (2014). Peptide-based vaccines for cancer therapy. Hum. Vaccines Immunother..

[54] Correale P., Botta C., Ciliberto D., Pastina P., Ingargiola R., Zappavigna S., Tassone P., Pirtoli L., Caraglia M., Tagliaferri P. (2016). Immunotherapy of colorectal cancer: New perspectives after a long path. Immunotherapy.

[55] Nakagawa H., Fujita M. (2018). Whole genome sequencing analysis for cancer genomics and precision medicine. Cancer Sci..

[56] Kamps R., Brandão R.D., Bosch B.J.V.D., Paulussen A.D.C., Xanthoulea S., Blok M.J., Romano A. (2017). Next-Generation Sequencing in Oncology: Genetic Diagnosis, Risk Prediction and Cancer Classification. Int. J. Mol. Sci..

[57] Mader S., Pantel K. (2017). Liquid Biopsy: Current Status and Future Perspectives. Oncol. Res. Treat..

[58] Burz C., Rosca A., Pop V.V., Buiga R., Aldea C., Samasca G., Silaghi C., Sur D., Lupan I., Pricopie A. (2019). Liquid biopsy challenge and hope in colorectal cancer. Expert Rev. Mol. Diagn..

[59] Normanno N., Cervantes A., Ciardiello F., De Luca A., Pinto C. (2018). The liquid biopsy in the management of colorectal cancer patients: Current applications and future scenarios. Cancer Treat. Rev..

[60] Brannon A.R., Vakiani E., Sylvester B.E., Scott S.N., McDermott G., Shah R.H., Kania K., Viale A., Oschwald D.M., Vacic V. (2014). Comparative sequencing analysis reveals high genomic concordance between matched primary and metastatic colorectal cancer lesions. Genome Biol..

[61] Lee S.Y., Haq F., Kim D., Jun C., Jo H.-J., Ahn S.-M., Lee W.-S. (2014). Comparative Genomic Analysis of Primary and Synchronous Metastatic Colorectal Cancers. PLoS ONE.

[62] Kim R., Schell M.J., Teer J.K., Greenawalt D.M., Yang M., Yeatman T.J. (2015). Co-Evolution of Somatic Variation in Primary and Metastatic Colorectal Cancer May Expand Biopsy Indications in the Molecular Era. PLoS ONE.

[63] Vignot S., Lefebvre C., Frampton G.M., Meurice G., Yelensky R., Palmer G., Capron F., Lazar V., Hannoun L., Miller V.A. (2015). Comparative analysis of primary tumour and matched metastases in colorectal cancer patients: Evaluation of concordance between genomic and transcriptional profiles. Eur. J. Cancer.

[64] Kovaleva V., Geissler A.L., Lutz L., Fritsch R., Makowiec F., Wiesemann S., Hopt U.T., Passlick B., Werner M., Lassmann S. (2016). Spa-tio-temporal mutation profiles of case-matched colorectal carcinomas and their metastases reveal unique de novo mutations in metachronous lung metastases by targeted next generation sequencing. Mol. Cancer.

[65] Ottaiano A., Caraglia M., Di Mauro A., Botti G., Lombardi A., Galon J., Luce A., D’Amore L., Perri F., Santorsola M. (2020). Evolution of Mutational Landscape and Tumor Immune-Microenvironment in Liver Oligo-Metastatic Colorectal Cancer. Cancers.

[66] Ottaiano A., Nasti G., Santorsola M., Altieri V., Di Fruscio G., Circelli L., Luce A., Cossu A.M., Scognamiglio G., Perri F. (2021). KRAS Mutational Regression Is Associated with Oligo-Metastatic Status and Good Prognosis in Metastatic Colorectal Cancer. Front. Oncol..

[67] Ottaiano A., Santorsola M., Caraglia M., Circelli L., Gigantino V., Botti G., Nasti G. (2021). Genetic regressive trajectories in colorectal cancer: A new hallmark of oligo-metastatic disease?. Transl. Oncol..

[68] Simanshu D., Nissley D.V., McCormick F. (2017). RAS Proteins and Their Regulators in Human Disease. Cell.

[69] Normanno N., Tejpar S., Morgillo F., De Luca A., Van Cutsem E., Ciardiello F. (2009). Implications for KRAS status and EGFR-targeted therapies in metastatic CRC. Nat. Rev. Clin. Oncol..

[70] Zhang X., Gureasko J., Shen K., Cole P.A., Kuriyan J. (2006). An Allosteric Mechanism for Activation of the Kinase Domain of Epidermal Growth Factor Receptor. Cell.

[71] Gilboa E. (1999). The Makings of a Tumor Rejection Antigen. Immunity.

[72] Heemskerk B., Kvistborg P., Schumacher T.N.M. (2012). The cancer antigenome. EMBO J..

[73] Schumacher T.N., Schreiber R.D. (2015). Neoantigens in cancer immunotherapy. Science.

[74] Perumal D., Imai N., Laganà A., Finnigan J., Melnekoff D.T., Leshchenko V.V., Solovyov A., Madduri D., Chari A., Cho H.J. (2020). Mutation-derived Neoantigen-specific T-cell Responses in Multiple Myeloma. Clin. Cancer Res..

[75] Sim M.J.W., Lu J., Spencer M., Hopkins F., Tran E., Rosenberg S.A., Long E.O., Sun P.D. (2020). High-affinity oligoclonal TCRs define effective adoptive T cell therapy targeting mutant KRAS-G12D. Proc. Natl. Acad. Sci. USA.

[76] Mazzaschi G., Madeddu D., Falco A., Bocchialini G., Goldoni M., Sogni F., Armani G., Lagrasta C.A., Lorusso B., Mangiaracina C. (2018). Low PD-1 Expression in Cytotoxic CD8(+) Tumor-Infiltrating Lymphocytes Confers an Immune-Privileged Tissue Micro-environment in NSCLC with a Prognostic and Predictive Value. Clin. Cancer Res..

[77] Pagès F., Mlecnik B., Marliot F., Bindea G., Ou F.-S., Bifulco C., Lugli A., Zlobec I., Rau T.T., Berger M.D. (2018). International validation of the consensus Immunoscore for the classification of colon cancer: A prognostic and accuracy study. Lancet.

[78] Li H., Durbin R. (2009). Fast and accurate short read alignment with Burrows-Wheeler transform. Bioinformatics.

[79] Li M.M., Datto M., Duncavage E.J., Kulkarni S., Lindeman N.I., Roy S., Tsimberidou A.M., Vnencak-Jones C.L., Wolff D.J., Younes A. (2017). Standards and Guidelines for the Interpretation and Reporting of Sequence Variants in Cancer: A Joint Consensus Rec-ommendation of the Association for Molecular Pathology, American Society of Clinical Oncology, and College of American Pathologists. J. Mol. Diagn..

[80] Yang H., Robinson P.N., Wang K. (2015). Phenolyzer: Phenotype-based prioritization of candidate genes for human diseases. Nat. Methods.

[81] Gámez-Cenzano C., Pino-Sorroche F. (2014). Standardization and Quantification in FDG-PET/CT Imaging for Staging and Restaging of Malignant Disease. PET Clin..

[82] Skougaard K., Nielsen D., Jensen B.V., Hendel H.W. (2013). Comparison of EORTC Criteria and PERCIST for PET/CT Response Evaluation of Patients with Metastatic Colorectal Cancer Treated with Irinotecan and Cetuximab. J. Nucl. Med..

[83] Mole R.H. (1953). Whole Body Irradiation—Radiobiology or Medicine?. Br. J. Radiol..

[84] Reynders K., Illidge T., Siva S., Chang J.Y., De Ruysscher D. (2015). The abscopal effect of local radiotherapy: Using immunotherapy to make a rare event clinically relevant. Cancer Treat. Rev..

